# Hsp90 is expressed and represents a therapeutic target in human oesophageal cancer using the inhibitor 17-allylamino-17-demethoxygeldanamycin

**DOI:** 10.1038/sj.bjc.6604855

**Published:** 2009-01-13

**Authors:** X Wu, A Wanders, P Wardega, B Tinge, L Gedda, S Bergstrom, L Sooman, J Gullbo, M Bergqvist, P Hesselius, J Lennartsson, S Ekman

**Affiliations:** 1 Unit of Oncology, Department of Oncology, Radiology and Clinical Immunology, Uppsala University, Uppsala SE-751 85, Sweden; 2 Department of Pathology, Uppsala University, Uppsala SE-751 85, Sweden; 3 Ludwig Institute for Cancer Research, Uppsala University, Uppsala SE-751 24, Sweden; 4 Unit of Biomedical Radiation Sciences, Department of Oncology, Radiology and Clinical Immunology, Rudbeck Laboratory, Uppsala University, Uppsala SE-751 85, Sweden

**Keywords:** oesophageal cancer, Hsp90, 17-AAG, EGF, IGF-1, radiation

## Abstract

Heat shock protein 90 (Hsp90) has been demonstrated to protect oncogenic variants of signalling molecules from degradation and may consequently serve as a therapeutic target for the treatment of oesophageal cancer for which adequate therapy is often lacking. We studied the expression of Hsp90 in tumour tissues of human oesophageal cancer and the impact of Hsp90 inhibition on oesophageal cancer cell lines using the drug 17-allylamino-17-demethoxygeldanamycin (17-AAG). Quantitative immunohistochemistry was performed on formalin-fixed paraffin-embedded tissues from patients with oesophageal cancer. In squamous cell carcinoma, a marked upregulation of Hsp90 could be noted in dysplastic epithelium and invasive cancer compared with normal epithelium. In adenocarcinoma, Hsp90 was expressed in neoplastic epithelium and also in normal non-neoplastic glands weakly. The inhibition of Hsp90 using 17-AAG led to a significant decrease in cell proliferation and viability in human oesophageal cancer cell lines. Using a clonogenic cell survival assay, Hsp90 inhibition significantly sensitised the cells for *γ*-photon irradiation. Heat shock protein 90 was found to be critical for proper signalling induced by both epidermal growth factor and insulin-like growth factor-1, in which the inhibition of signalling by 17-AAG correlated with the observed reduction in cell proliferation and viability. These results showed that Hsp90 was selectively expressed in oesophageal cancer tissue compared with the corresponding normal tissue, and the inhibition of Hsp90 resulted in decreased proliferation and viability as well as radiosensitisation of oesophageal cancer cells. Heat shock protein 90 represents a potential therapeutic target in the treatment of patients with oesophageal cancer, alone or in combination with radiotherapy.

Oesophageal cancer is histopathologically divided into adenocarcinomas and squamous cell carcinomas, but standard treatment is similar, including surgery, irradiation and chemotherapy. Despite advances in surgical techniques and treatment, the prognosis of oesophageal cancer remains poor with very few long-term survivors (Enzinger and Mayer, 2003).

Heat shock protein 90 (Hsp90) is an abundant protein that functions as a chaperone, thus preventing protein aggregation and helping denatured proteins to refold in an ATP-dependent fashion (Panaretou *et al*, 1998; Meyer *et al*, 2003). 17-Allylamino-17-demethoxygeldanamycin (17-AAG) is an inhibitor of Hsp90 that binds to the nucleotide-binding site and thereby promotes the release of the client proteins, which may then be degraded (Schneider *et al*, 1996). In many cancer cells, Hsp90 functions to protect and stabilise overexpressed or mutated signal transduction proteins, thus indirectly promoting cell growth and survival (Neckers and Ivy, 2003). A role for Hsp90 in the regulation of apoptosis has also been suggested, acting both in an antiapoptotic and pro-apoptotic manner (Pandey *et al*, 2000; Nieto-Miguel *et al*, 2007).

Hanahan and Weinberg (2000) have suggested that the transformation of a normal cell into a cancer cell requires six essential alterations in cell physiology that collectively dictate malignant growth: self-sufficiency in growth signals, insensitivity to growth-inhibitory signals, evasion of apoptosis, limitless replicative potential, sustained angiogenesis and tissue invasion and metastasis (Hanahan and Weinberg, 2000). In a review by Xu and Neckers (2007), it is pointed out that because many Hsp90 client proteins play significant roles in each of these alterations, the therapeutic potential of targeting Hsp90 may be best appreciated by considering the possibility of simultaneously targeting the six hallmarks of a cancer cell.

In this report, we investigate the expression of Hsp90 in human oesophageal cancer and the importance of Hsp90 for oesophageal cancer cell proliferation and sensitivity towards *γ*-photon radiation. Our results show that Hsp90 is abundantly expressed in human tumours of oesophageal cancer as well as in human oesophageal cancer cell lines. 17-Allylamino-17-demethoxygeldanamycin effectively inhibits cell proliferation of oesophageal carcinoma cell lines, indicating the presence of mutated or overexpressed signalling proteins that are protected from degradation by Hsp90. In addition, Hsp90 inhibition increases the sensitivity towards *γ*-photon radiation. The effect of 17-AAG on proliferation and radiosensitivity is correlated with the ability of 17-AAG to downregulate growth factor-mediated signal transduction.

## Materials and methods

### Cell culture, antibodies and reagents

Kyse30, -70, -140, -150, -180, -410, -450, -510 and -520 cell lines derived from oesophageal squamous cell carcinoma were purchased from Deutsche Sammlung von Mikroorganismen und Zellkulturen (Braunschweig, Germany). Cells were cultured in RPMI-1640 medium (Sigma, St Louis, MO, USA) supplemented with 10% foetal bovine serum, 50 U ml^−1^ penicillin, 100 *μ*g ml^−1^ streptomycin and 2 mM L-glutamine in a 37°C incubator with humidified atmosphere and 5% CO_2_. Antibodies used were anti-epidermal growth factor receptor (EGFR), anti-insulin-like growth factor-1 (IGF)-1R*β* and anti-Akt1/2 rabbit polyclonal antibodies (Santa Cruz Biotechnology, Santa Cruz, CA, USA), anti-Hsp90 mouse monoclonal antibodies (Biosite, San Diego, CA, USA), anti-phosphotyrosine mouse monoclonal antibodies PY99 (Santa Cruz), phosphospecific anti-Erk and phosphospecific anti-Akt antibodies (Cell Signalling Technology, Beverly, MA, USA) and anti-*β*-actin mouse monoclonal antibodies (Sigma) and horseradish peroxidase (HRP)-conjugated anti-mouse IgG or anti-rabbit IgG secondary antibodies (Amersham Biosciences, Piscataway, NJ, USA). Anti-Erk2 rabbit serum was from the Ludwig Institute, Uppsala, Sweden. Gefitinib was from Biaffin GmbH & Co. KG (Kassel, Germany), 17-AAG from AG Scientific Inc. (San Diego, CA, USA), EGF from Chemicon International Inc. (Temecula, CA, USA) and IGF-1 from R&D Systems Inc. (Minneapolis, MN, USA).

### Patients

All patients with oesophageal carcinoma admitted to the Department of Oncology, University Hospital, Uppsala, Sweden, during 1990–2000 were included. The study was reviewed and approved by the research ethics committee, Uppsala University, Uppsala, Sweden. A total of 126 patients were included; however, only formalin-fixed paraffin-embedded tissues from 81 patients could be retrieved and thereby included in the study. The clinical parameter investigated was survival.

### Immunohistochemical analysis

Heat shock protein 90 immunohistochemical (IHC) staining was performed in 81 archival paraffin-embedded tumour samples. The immunostaining was performed according to the method published by Dreilich *et al* (2006). Samples with a known expression of Hsp90 (HeLa cells) were used as positive control. Sections were incubated with the primary antibody (Hsp90 Ab-2 (JPB24), Santa Cruz) and an automated immunohistochemical system from Ventana (Benchmark; Ventana Medical Systems, Tuscon, AZ, USA) was employed according to the manufacturer's recommendations. Immunostained tissues were annotated by an experienced gastrointestinal pathologist according to the criteria used in the Swedish human protein atlas program (http://www.proteinatlas.org/annotdesc.php). The extent of positive tumour cells was scored using a three-grade scale: (1) <25% positive tumour cells, (2) 25–75% positive cells and (3) >75% of tumour cells staining positively. The intensity of immunoreactivity in tumour cells was evaluated using a four-grade scale: faint (1), weak (2), moderate (3) and strong (4). The extent and intensity scores were used as a basis for grading immunoreactivity in oesophageal cancer cells. In addition, the subcellular localisation was evaluated: membranous, cytoplasmic or nuclear positivity.

### Statistical analysis

Heat shock protein 90 was evaluated as a dichotomous variable. The expression of Hsp90 was according to above. Survival was estimated using the Kaplan–Meier product limit method, with univariate analysis being performed using a log-rank test. Cox regression analysis was performed to investigate if certain continuous factors had a significant effect on survival. Throughout the study, a 5% significance level was used.

### Proliferation assay

Duplicates of 50 000 cells suspended in complete medium were seeded into the wells of 12-well plates (Fisher Scientific, Pittsburgh, PA, USA). After the cells were attached, 17-AAG or gefitinib was added to each well at the designated concentration. The concentration of DMSO in the control and treatment wells was 0.1%. Cells were trypsinised and counted in a cell counter (Beckman Coulter, Fullerton, CA, USA) after the indicated periods of time. The number of cells in untreated control wells was defined as 100%.

### Apoptosis assay

Kyse70 and Kyse450 cells were plated, returned to the incubator for 24 h and then treated with 17-AAG or DMSO for another 24 h and exposed to irradiation. After that, the drug was removed and fresh medium added, and the cells were incubated for another 48 h. Both floating and attached cells were collected by centrifugation. Apoptosis analysis was performed according to the manufacturer's instructions (Annexin V-FITC Apoptosis detection kit; R&D Systems Inc.). Results for early and late apoptosis were added together as total amount of apoptosis (Bisht *et al*, 2003; Martins *et al*, 2008) (I add the reference, but the format is stange).

### Irradiation and clonogenic survival assay

Cells were seeded into T-25 flasks at a density of 2 × 10^5^ per flask and allowed to grow for 24 h in a humidified atmosphere with 5% CO_2_ in a 37°C incubator. After attaining sufficient growth, the designated concentration of 17-AAG or gefitinib was added to the growth media and the cells were incubated for 24 h. Thereafter, the cells were irradiated with 2, 4, 6 or 8 Gy *γ*-photons from a ^137^Cs source (1.3 Gy min^−1^; Gammacell 40 Exactor; MDS Nordion, Ottawa, ON, Canada). Following irradiation, cells were trypsinised and plated onto 10-cm tissue culture dishes at various cell densities for clonogenic cell survival. Cells were incubated for 10–11 days, fixed with ethanol and stained by haematoxylin. Clonogenic cells were defined as those able to form a colony of atleast 50 cells. Radiosensitivity was quantified as area under curve (AUC), and the effect of 17-AAG on radiosensitivity is expressed as sensitiser enhancement ratio (SER), defined as AUC_control_/AUC_treated_.

### Immunoprecipitation and western blot analysis

For ligand stimulation and 17-AAG treatment, subconfluent Kyse70 and Kyse450 cells were treated with 0.5, 1 and 1.5 *μ*M 17-AAG for 24 and 48 h as indicated in starvation medium containing 0.1% foetal bovine serum at 37°C. Next, the cells were stimulated with 100 ng ml^−1^ EGF or IGF-1 for 5 min and washed with cold phosphate-buffered saline before lysis. Cell lysates were prepared according to Lennartsson *et al* (2006). Briefly, total protein concentration was determined using the BCA Protein Assay Kit (Pierce, Rockford, IL, USA). Total cell lysates were submitted to SDS–polyacrylamide gel electrophoresis. For immunoprecipitation, antibodies against Hsp90 were added to each lysate at a concentration of 1 *μ*g ml^−1^. Protein G beads were added to collect immunocomplexes. After washing beads, samples were boiled in reducing sample buffer and subjected to SDS–polyacrylamide gel electrophoresis. Separated proteins were electrotransferred to polyvinylidene fluoride membranes (Millipore, Billerica, MA, USA). Membranes were blocked using 5% BSA, incubated with primary antibody overnight at 4°C and then incubated with HRP-conjugated anti-mouse or anti-rabbit IgG antibodies (both from Amersham Biosciences). Proteins were visualised using an ECL western blotting detection system from Roche Applied Science on a cooled charge-coupled device camera (Fuji, Minami-Ashigata, Japan). Before reprobing, the membranes were stripped with 0.4 M NaOH for 10 min at room temperature, blocked and incubated with the corresponding antibody.

## Results

### Hsp90 expression in tumours and cell lines of human oesophageal cancer

**Figure 1:**
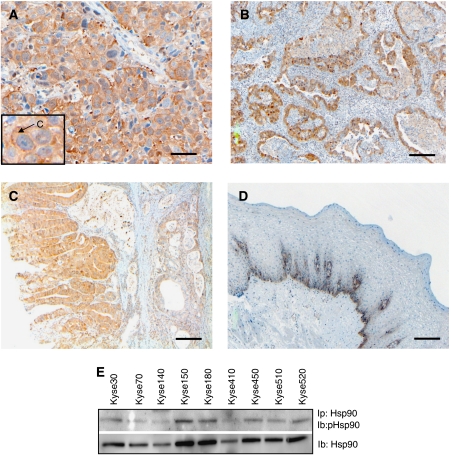
Hsp90 is expressed in tumours and cell lines of human oesophageal cancer. Protein expression of Hsp90 was evaluated in tumours from 81 oesophageal cancer patients using an immunohistochemical staining procedure. Representative stainings of tumour tissues are shown in (**A**) (squamous cell carcinoma), (**B**) (adenocarcinoma) and (**C**) (dysplasia), whereas staining of normal oesophageal epithelium is demonstrated in (**D**). A predominant cytoplasmic staining was observed in the oesophageal tumours, as indicated by an arrow in the box of figure (**A**). Scale bar=50 *μ*m (**A**) or 100 *μ*m (**B**–**D**). (**E**) Hsp90 was immunoprecipitated (Ip) and the membrane was subjected to immunoblotting (Ib) with antibodies against phosphotyrosine (upper panel) or Hsp90 (lower panel). The experiment was repeated atleast three times, a representative result is shown.

To evaluate Hsp90 as a potential target for therapy in oesophageal cancer, the protein expression of Hsp90 was investigated in tumours from 81 oesophageal cancer patients, 53 squamous cell carcinomas and 28 adenocarcinomas, using IHC staining. From the original 85 patients included, four patients had to be excluded due to uncertain histology. For both squamous cell carcinomas and adenocarcinomas, the majority of tumours were found to express Hsp90 at weak-to-moderate intensity levels (44 and 56%, respectively) (Figure 1A and B, and Table 1), but squamous cell carcinomas displayed a higher fraction of moderately expressing tumours (23%) compared with adenocarcinomas (4%). Furthermore, adenocarcinomas showed a higher extent of weak staining (40%) compared with squamous cell carcinomas (30%). There was also less difference in staining intensity between tumour and normal tissue in adenocarcinomas compared with squamous carcinomas. Normal oesophageal squamous epithelium had only a faint Hsp90 expression of the basal cell layer (Figure 1D). For both squamous and glandular epithelium, a low-to-moderate staining was observed in dysplastic epithelium (Figure 1C).

Preferentially, cytoplasmic staining was observed, representing 70% of the tumours (57 out of 81). Nuclear staining was observed in 2.5% of the tumours (2 out of 81), whereas 13.6% (11 out of 81) displayed both cytoplasmic and nuclear localisation (Figure 1A). The importance of these findings remains to be elucidated, but the dominating cytoplasmic Hsp90 staining is consistent with a function for Hsp90 in stabilising signal transduction molecules in this cellular compartment.

Analysing the protein expression of Hsp90 in human oesophageal cancer cell lines, we found a strong expression in all nine cell lines analysed (Figure 1E). This is consistent with earlier reports demonstrating abundant Hsp90 expression in various tumour cells (Ferrarini *et al*, 1992; Liu *et al*, 1999; Pick *et al*, 2007). We selected Kyse70 and -450 for further investigations.

### Hsp90 as a prognostic factor in oesophageal cancer

**Figure 2:**
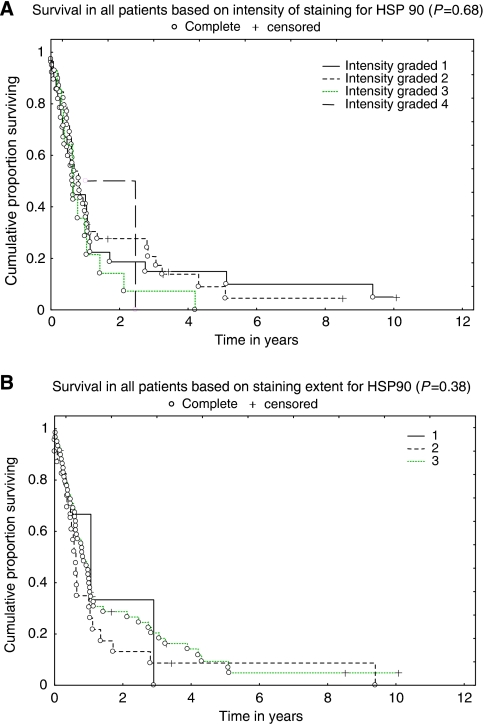
Hsp90 is not a prognostic factor in oesophageal cancer. The expression of Hsp90 in oesophageal tumours was evaluated in relation to survival using the Kaplan–Meier product limit method, with univariate analysis being performed using a log-rank test. Both (**A**) intensity and (**B**) the extent of staining are shown.

The expression of Hsp90 in oesophageal tumours was evaluated in relation to survival using the Kaplan–Meier product limit method, with univariate analysis being performed using a log-rank test. Both intensity and the extent of staining were evaluated. No significant correlation to survival was observed, neither for intensity (*P*=0.68) (Figure 2A) nor for extent (*P*=0.38) (Figure 2B). Type of histology, squamous cell carcinoma or adenocarcinoma, did not make any difference (*P*=0.50 and *P*=0.93 for intensity, respectively; data not shown). Detailed characteristics of patients and IHC stainings are shown in Table 1.

### Hsp90 blockade by 17-AAG inhibits cell proliferation and survival

**Figure 3:**
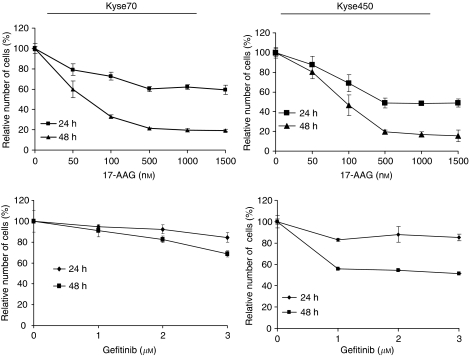
17-AAG inhibits proliferation of oesophageal cancer cell lines. Kyse70 (left) and Kyse450 (right) cells were treated with the indicated concentrations of 17-AAG (upper panels) or gefitinib (lower panels) for the indicated periods of time, and cells were then counted. Relative number of cells as % of control was calculated and shown. Each data point had at least three repeats.

Considering the fact that Hsp90 was expressed in all oesophageal tumours analysed, we investigated the role of Hsp90 in cell proliferation and survival by performing specific inhibition using 17-AAG. Both Kyse70 and Kyse450 cell lines displayed a dose- and time-dependent reduction in cell proliferation (Figure 3, upper panels). A maximal reduction of proliferation was achieved after treatment with 500 nM 17-AAG for 48 h, where Kyse70 and Kyse450 showed 80 and 84% inhibition, respectively.

Epidermal growth factor receptor is often abundantly expressed in oesophageal cancer (Ozawa *et al*, 1987; Mukaida *et al*, 1991) and therefore we tested the ability of gefitinib to inhibit cell proliferation. At 48 h of treatment with 3 *μ*M gefitinib, there was a 31% reduction of proliferation for Kyse70 and 49% for Kyse450 (Figure 3, lower panels). The gefitinib concentrations chosen correspond to the plasma concentrations of gefitinib in treated patients (Sharma *et al*, 2007). Using 3 *μ*M gefitinib for 48 h, a significantly lower inhibition of proliferation was seen compared with 500 nM 17-AAG for 48 h (*P*<0.05 for both Kyse70 and Kyse450 cells).

**Figure 4:**
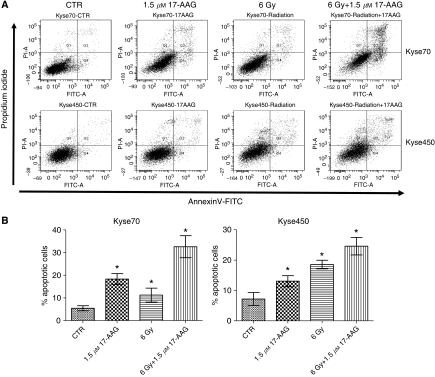
Effects of 17-AAG on apoptosis of oesophageal cancer. Subconfluent Kyse70 and Kyse450 cells were treated with 1.5 *μ*M 17-AAG, exposed to irradiation (6 Gy), or treated with 17-AAG before exposure to irradiation. Cells were harvested at 48 h after exposure to irradiation and apoptosis was analysed by flow cytometry using Annexin-V/PI detection. Results of early and late apoptosis were added together to calculate the total amount of apoptosis. (**A**) Graphical representation of apoptosis for all treatments of Kyse70 and Kyse450 cells. Irradiation, 17-AAG and the combination could induce apoptosis for both cell lines. Combination of irradiation and 17-AAG induced a higher degree of apoptosis in Kyse70 compared with Kyse450 cells. (**B**) Bar graph of all conditions. Irradiation combined with 17-AAG resulted in an additional increase of apoptosis in both cell lines. Statistical significance was established by *t*-test. ^*^P<0.05; this treatment showed a difference compared with other treatments and control.

The effect of 17-AAG on apoptosis induction was studied in the Kyse cell lines. As depicted in Figure 4A and B, treatment with 17-AAG alone resulted in significantly (*P*<0.05) increased rates of apoptosis, with 18% apoptosis for Kyse70 and 13% for Kyse450 compared with control. These results showed that 17-AAG can induce apoptotic cell death in oesophageal cancer cells.

### Hsp90 inhibition can radiosensitise oesophageal cells

**Figure 5:**
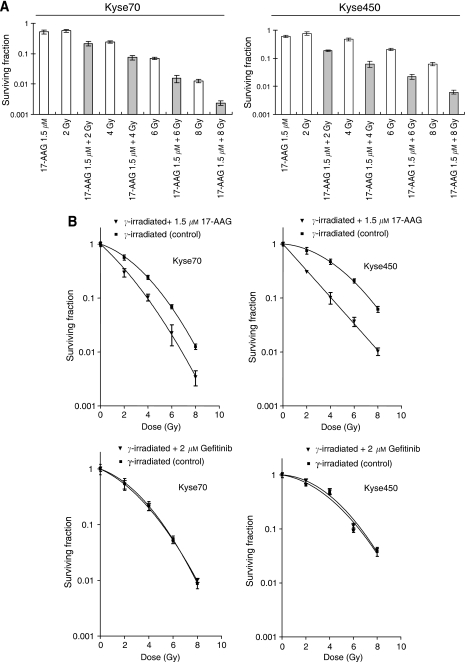
17-AAG radiosensitises oesophageal cancer cell lines. Clonogenic cell survival of Kyse70 and Kyse450 cells following 17-AAG treatment (1.5 *μ*M for 24 h) and/or *γ*-photon irradiation (2, 4, 6 or 8 Gy). (**A**) Shows survival fraction as a function of 17-AAG, *γ*-dose and their combination. (**B**) Shows survival plots as a function of *γ*-dose in the absence or presence of 17-AAG or gefitinib at the indicated concentrations. The sensitiser enhancement ratio (SER) for 17-AAG was calculated to be 1.5 and 2.1 for Kyse70 and Kyse450, respectively. Survival data were fitted to a linear quadratic curve fit, *S*=exp(−*αD*−*βD*^2^) where *D* is the dose in Gy and *α* and *β* are fitting parameters, *n*⩾6, error bars represent standard deviation. All 17-AAG treatment groups had *P*-values < 0.05.

Earlier studies have indicated that Hsp90 inhibition may radiosensitise tumour cells (Camphausen and Tofilon, 2007). To address the same issue in oesophageal cancer cells, we performed clonogenic cell survival assays to measure *in vitro* cell killing as a function of radiation dose. Kyse70 and Kyse450 cells were irradiated with 2, 4, 6 or 8 Gy *γ*-photons in the presence or absence of 17-AAG and cell survival was studied. Exposure to 17-AAG alone resulted in 51% cell survival for Kyse70 and 61% cell survival for Kyse450 (Figure 5A). Cell survival after 2, 4, 6 and 8 Gy irradiation for Kyse70 was 56, 24, 6.9 and 1.2%, respectively. Theoretically, the expected additive cell survival of Kyse70 after exposure to drug and *γ*-photons (0.51 × survival*γ*) should be approximately 28, 12, 3.5 and 0.6%. However, the achieved cell survival after combination was less; 21, 7.5, 1.5 and 0.2% for 2, 4, 6 and 8 Gy, respectively (Figure 5A). For Kyse450 this effect was even more apparent. At 2, 4, 6 and 8 Gy, the expected additive survival should be 46, 29, 13 and 3.7%, whereas the achieved survival was 18, 6.2, 2.2 and 0.7% (Figure 5A). Figure 5B (upper panels) showed the survival curves of Kyse70 and Kyse450 after *γ*-photon irradiation or *γ*-photon combined with 17-AAG treatment. As irradiated and 17-AAG-treated cells were normalised to 17-AAG-treated controls, the figure only reflects the radiosensitising effect. We observed that 17-AAG sensitised the radioresistant Kyse450 cells more than the radiosensitive Kyse70 cells, and the obtained SERs were 2.1 and 1.5, respectively. Importantly, gefitinib treatment was not able to radiosensitise either Kyse70 or Kyse450 cells (Figure 5B, lower panels).

Apoptosis is a mode of cell death in response to radiation. As depicted in Figure 4A and B, radiation (6 Gy) significantly increased apoptosis for both cell lines compared with control (*P*<0.05). Apoptosis rate for Kyse70 cells was approximately 12% and for Kyse450 cells was approximately 18% at 6 Gy. When 17-AAG was combined with radiation, the apoptosis rate increased to 32% for Kyse70 and 24% for Kyse450, significantly higher rates compared with radiation or 17-AAG treatment alone (*P*<0.05).

### 17-AAG inhibits pro-survival signalling pathways

**Figure 6:**
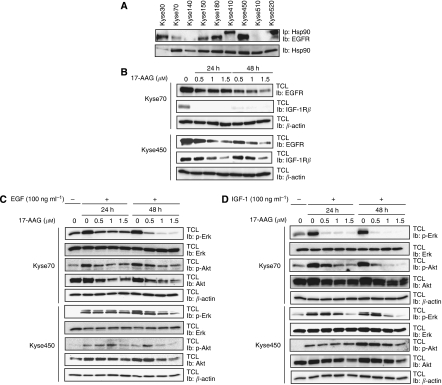
Hsp90 interacts with the EGF receptor and 17-AAG treatment downregulates EGF and IGF-1 receptor signalling. Cells (Kyse70 and Kyse450) were grown to confluence and then lysed. (**A**) Hsp90 was immunoprecipitated (Ip) and the membrane was subjected to immunoblotting (Ib) with antibodies against EGF receptor. (**B**) Kyse70 and Kyse450 cells were treated with 17-AAG at increasing concentrations for 24 or 48 h. Total cell lysate (TCL) was prepared and the level of EGF and IGF-1 receptor was analysed by immunoblotting (Ib). (**C** and **D**) Kyse70 and Kyse450 cells were grown to subconfluence, starved and then treated with indicated concentrations of 17-AAG for 24 or 48 h. After stimulation with EGF or IGF-1 for 5 min, cell lysates were prepared and phospho-Erk and phospho-Akt levels were analysed by immunoblotting as indicated ((**C**) EGF stimulation and (**D**) IGF-1 stimulation). *β*-Actin was used as loading control for each blot. The experiment was repeated atleast three times, a representative result is shown.

Epidermal growth factor receptor is often overexpressed in patients with oesophageal cancer, suggesting an important role for it in the development of this disease (Sarbia *et al*, 2007; Wang *et al*, 2007; Wei *et al*, 2007). All Kyse cell lines were found to express EGFRs, whereas HER2 was expressed only in Kyse410 (data not shown). Furthermore, we observed an interaction between the EGFR and Hsp90 in co-immunoprecipitation experiments (Figure 6A). In addition, we could detect tyrosine phosphorylation of Hsp90 in all of the cell lines (Figure 1E, upper panel). Notably, the level of Hsp90 phosphorylation was not sensitive to either 17-AAG or gefitinib treatment (data not shown). Epidermal growth factor receptor was downregulated by 17-AAG treatment in a dose- and time-dependent manner in both Kyse70 and Kyse450 cell lines (Figure 6B). Moreover, we found that the EGF-induced activation of the downstream signalling proteins Erk and Akt was also inhibited by 17-AAG (Figure 6C). Interestingly, the level of Akt protein was sensitive to 17-AAG treatment, whereas the inhibition of Erk signalling was not due to changes in total protein levels. As Hsp90 is known to have many different client proteins, we wanted to investigate whether other pro-survival signalling pathways were affected in a similar way by the inhibition of Hsp90. The IGF-1 signalling cascade is a well-established and ubiquitously expressed pro-survival pathway, and the IGF-1 receptor has been proposed to confer survival signals to a wide range of tumour cells (Liu *et al*, 2002; Stromberg *et al*, 2006). As the IGF-1 receptor is also expressed in Kyse70 and Kyse450 cells, we wanted to investigate whether 17-AAG could also downregulate this survival pathway. As can be seen in Figure 6B, we found that 17-AAG efficiently downregulated the IGF-1 receptor. In concurrence, IGF-1-induced Erk and Akt activation was also potently inhibited by 17-AAG treatment (Figure 6D).

## Discussion

Several reports have indicated the important role of Hsp90 in carcinogenesis (Isaacs *et al*, 2003; Citri *et al*, 2004; Zhang and Burrows, 2004). However, studies in oesophageal cancer are sparse and to elucidate this issue further, this study sought to evaluate the importance of Hsp90 as the primary target for novel therapy using 17-AAG in oesophageal cancer.

Heat shock protein 90 was found to be expressed in all investigated oesophageal tumours (81 patients), whereas normal oesophageal epithelium expressed no or very low levels of the protein. The selective expression in this tumour showed promise for further evaluation of Hsp90 as a target for therapy in oesophageal cancer. The observed data are in contrast to the study by Faried *et al* (2004), who demonstrated an expression of Hsp90 in only 50% of the tumours (123 cases). The reason for the observed differences is not clear. All cases in the study by Faried *et al* (2004) were squamous cell carcinomas, whereas our study included both squamous cell carcinomas and adenocarcinomas, however, the squamous cancers dominating (65%) and all demonstrating a clear expression of Hsp90. These contradictory results might be explained by differences in stages of disease, treatment modalities as well as different populations, which may have different expressions of oncogenic proteins as seen for HER2 in oesophageal cancer (Lam *et al*, 1998; Dreilich *et al*, 2006). Furthermore, a relatively higher expression of Hsp90 was found in squamous cell carcinomas compared with adenocarcinomas, a finding that has never been reported before. Both squamous and glandular epithelium displayed a low-to-moderate staining in dysplastic epithelium. A similar finding was reported by Ito *et al* (1998), who demonstrated the expression of Hsp90 already in dysplastic lesions of squamous epithelium of the tongue. This is consistent with the notion that early in tumorigenesis, the incipient tumour experiences ‘oncogenic stress’, which evokes a DNA damage response network that delays or prevents cancer (Bartkova *et al*, 2005). This ‘oncogenic stress’ results in the inducible expression of heat shock proteins that assist in the recovery from stress either by repairing damaged proteins (protein refolding) or by degrading them, thus restoring protein homoeostasis and promoting cell survival (Jolly and Morimoto, 2000). In Barrett's oesophagus, a reduced expression of heat shock proteins in metaplasia followed by a significant increase in expression during progression was shown (Doak *et al*, 2004; Ostrowski *et al*, 2007). Thus, the expression of heat shock proteins, including Hsp90, presents potential as a prognostic tool to predict the aggressiveness of oesophageal carcinomas.

A preferential cytoplasmic localisation of Hsp90 was found in the tumours (70%), whereas nuclear staining was detected in only 2.5% of the cases. These results are contradictory to the data reported earlier in malignant tumours, in which Burkitt *et al* (2007) observed nuclear localisation in 40% (10/25) of prostatic adenocarcinomas, but in none of the non-malignant specimens. In pancreatic malignancy, 14 out of 15 (93%) had nuclear staining, whereas none had nuclear staining in non-malignant tissue. A change in localisation of Hsp90 in tumours would be predicted to change the pattern of bound Hsp90 clients and may contribute to the specificity of 17-AAG for tumour cells. Furthermore, a study in human breast cancer demonstrated a positive correlation between strong nuclear staining for Hsp90 and high MHC class I expression, leading to the hypothesis that tumour cells with a high MHC class I expression may escape apoptosis by a mechanism involving increased nuclear Hsp90 (Gebhard *et al*, 1999). The importance of predominant cytoplasmic staining in oesophageal cancer remains to be determined, but may reflect an increased binding of cytoplasmic Hsp90 client proteins.

In this study, we observed Hsp90 expression in all oesophageal cancer cell lines investigated. Moreover, we detected tyrosine phosphorylation of Hsp90, which recently has been connected to Hsp90 functions in vascular endothelial growth factor receptor-2 signalling (Duval *et al*, 2007). However, the functional consequence of Hsp90 tyrosine phosphorylation in our model system is not understood and warrants further investigation. Studies on breast cancer patients have shown that a high Hsp90 expression was found to be associated with decreased survival in breast cancer (Pick *et al*, 2007). In a study by Lebret *et al* (2007), the authors found that the loss of expression of Hsp90 was correlated with a worse clinical outcome in patients with bladder carcinoma. Regarding oesophageal cancer, Faried *et al* (2004) studied the prognostic importance of Hsp60 and Hsp90 in oesophageal cancer patients and found no correlation with prognosis for Hsp90, whereas Hsp60 expression correlated with favourable prognosis. This is consistent with the results of this study, in which no significant correlation was found between Hsp90 expression and survival, neither for squamous cell carcinoma nor for adenocarcinoma, as all tumours analysed displayed robust Hsp90 staining regardless of the disease outcome. In contrast, normal tissue displayed no or weak staining, indicating that Hsp90 may represent a tumour-selective therapeutic target.

The inhibition of Hsp90 using 17-AAG in two oesophageal cell lines (Kyse70 and Kyse450) resulted in an inhibition of cell proliferation in a dose- and time-dependent manner. This is consistent with studies on other tumour types, for example, cervical carcinoma, lymphoma and thyroid cancer, in which 17-AAG treatment showed antiproliferative activity (Park *et al*, 2003; Schumacher *et al*, 2007; Schwock *et al*, 2008). Furthermore, we found that treatment with 17-AAG sensitises oesophageal cancer cells to *γ*-photon radiation. A further observation was that SER was larger for Kyse450 than for Kyse70, 2.1 and 1.5, respectively. It should be noted that Kyse450 is more radioresistant than Kyse70, and our results are thus consistent with reports that 17-AAG has a better sensitisation effect on radioresistant cell lines (Russell *et al*, 2003; Machida *et al*, 2005). Inhibition of the EGFR, which is highly expressed in these cell lines and frequently expressed at high levels in oesophageal tumours, did not influence the radiosensitivity, although we could observe an effective inhibition of EGFR signalling after gefitinib treatment (data not shown).

The inhibition of Hsp90 has been shown to impair EGF-mediated signalling in gastric cancer cells (Lang *et al*, 2007a). Inhibitory effects on other growth factor signals have also been implicated, for example, IGF-1R (Lang *et al*, 2007b) and Kit (Bauer *et al*, 2006). We found that the reduced proliferation and radiosensitising effects of Hsp90 inhibition in oesophageal cancer cell lines correlated with a decrease in growth factor-induced signalling. In Kyse410 and Kyse520 cells we noticed that the co-immunoprecipitating EGFR appears as a band of higher molecular weight than expected (Figure 6A). Elucidation of the reason for this apparent increase in molecular weight warrants further investigations, but possible explanations include mutations (insertion) or a high level of post-translational modification.

When we analysed the effect of 17-AAG on the receptors for EGF and IGF-1 we found that 17-AAG treatment downregulated both the receptors in a time- and dose-dependent manner. In addition, the downstream signalling molecules Akt and Erk were inhibited. Given that the reduced Erk phosphorylation was not due to decreased protein levels it is likely a secondary effect caused by receptor downregulation in response to 17-AAG. In contrast, reduced levels of phosphorylated Akt were reflected in lower levels of total Akt protein; thus, activated Akt may require Hsp90 activity to avoid degradation. However, the inactivation or downregulation of upstream Akt regulators is also likely to contribute to the reduced Akt phosphorylation in the presence of 17-AAG treatment.

The radiosensitising effect of 17-AAG may potentially be due to its ability to affect a range of signalling components simultaneously, for example both EGF and IGF-1 signalling networks as described above. Moreover, we observed that Hsp90 inhibition blocks serum-induced tyrosine phosphorylation of several proteins of unknown identity (data not shown). The resulting combinatorial effect may be of significance as more selective compounds such as gefitinib, although effectively preventing EGF signalling, were not able to radiosensitise the cells in our study.

In conclusion, our data suggest that Hsp90 represents a novel target in oesophageal cancer therapy both in a single-drug and a radiosensitising setting, conceivably by its ability to impede with a large spectrum of cellular components controlling proliferative and antiapoptotic signalling pathways.

## Figures and Tables

**Table 1:** Characteristics of patients and immunohistochemical stainings

			**Staining intensity**				**Extent of staining**		
	**No. of patients**	**Survival (days)**	**1**	**2**	**3**	**4**	**1**	**2**	**3**
*Gender*									
Male	59	433	18 (30%)	27 (46%)	13 (22%)	1 (2%)	2 (3%)	16 (27%)	41 (69%)
Female	26	731	10 (42%)	12 (50%)	1 (4%)	1 (4%)	1 (4%)	7 (27%)	18 (69%)
									
*Histology*									
Squamous cell	59	556	17 (30%)	25 (44%)	13 (23%)	2 (4%)	1 (2%)	14 (24%)	44 (75%)
Adenocarcinoma	25	452	10 (40%)	14 (56%)	1 (4%)	0 (−)	2 (8%)	8 (32%)	15 (60%)
Other	1	—	—	—	—	—	—	—	—
									
Stage									
Localised	27	251	11 (41%)	10 (37%)	6 (22%)	0 (0%)	1 (4%)	8 (30%)	18 (66%)
Advanced	58	651	17 (30%)	29 (52%)	8 (14%)	2 (4%)	2 (3%)	15 (26%)	41 (71%)
									
Localisation of tumour									
Upper	9	686	5 (63%)	2 (25%)	1 (13%)	0 (0%)	1 (11%)	4 (44%)	4 (44%)
Middle	26	594	9 (36%)	15 (60%)	1 (4%)	0 (0%)	1 (4%)	6 (23%)	19 (73%)
Lower	42	372	11 (26%)	19 (45%)	10 (24%)	2 (5%)	1 (2%)	11 (26%)	30 (71%)
